# Acute hemorrhagic leukoencephalitis (Weston-Hurst syndrome) in a patient with relapse-remitting multiple sclerosis

**DOI:** 10.1186/s12974-015-0398-1

**Published:** 2015-09-17

**Authors:** Özlem Yildiz, Refik Pul, Peter Raab, Christian Hartmann, Thomas Skripuletz, Martin Stangel

**Affiliations:** Department of Neurology, Hannover Medical School, Hannover, Germany; Department of Neuroradiology, Hannover Medical School, Hannover, Germany; Department of Neuropathology, Hannover Medical School, Hannover, Germany

**Keywords:** Fulminant demyelinating disease, Multiple sclerosis, Acute hemorrhagic leukoencephalitis, Weston-Hurst syndrome, Acute necrotizing encephalitis

## Abstract

Acute hemorrhagic leukoencephalitis is a fulminant demyelinating disease and commonly considered as a rare and severe variant of acute disseminated encephalomyelitis. Here, we report the clinical, magnetic resonance imaging, and brain biopsy findings of a 35-year-old female with relapsing-remitting multiple sclerosis, who developed acute hemorrhagic leukoencephalitis. Magnetic resonance imaging revealed symmetrical hemorrhagic lesions in the basal ganglia including the thalami. Disease progression was consistent with acute hemorrhagic leukoencephalitis with rapid deterioration of consciousness and seizures. Besides hemorrhage, infiltration of neutrophils was detected in brain biopsy.

Acute hemorrhagic leukoencephalitis, also known as Weston-Hurst syndrome, is an excessive immunological response of unknown etiology. So far, an association with multiple sclerosis has not been reported. The present case raises the question, whether acute hemorrhagic leukoencephalitis is a specific hyperacute form of acute disseminated encephalomyelitis, a severe and unspecific form of an immune response in the central nervous system, or belongs to the spectrum of tumefactive multiple sclerosis.

## Introduction

Acute hemorrhagic leukoencephalitis (AHLE) was first described by Weston Hurst in 1941. It is commonly considered as a rare and severe variant of acute disseminated encephalomyelitis (ADEM) and represents a rather fulminant course of this disease [1]. AHLE is characterized by an acute onset and rapidly progressive inflammation with symmetrical, multifocal brain lesions associated with acute edematous necrosis and hemorrhage. This rare disease typically affects young adults and is often associated with preceding (1–4 weeks) respiratory tract infections [1, 2]. Multiple sclerosis (MS) is a chronic disease of the central nervous system (CNS) whose neuropathological hallmarks are inflammation and demyelination leading to axonal degeneration [3]. The evolution of MS lesions depends upon whether the course of the disease is relapsing-remitting or chronic [3]. Four different neuropathological patterns of demyelinating lesions have been reported, but in none of these lesions, hemorrhage due to microvascular damage or necrosis can be found [3, 4].

## Case presentation

**Fig. 1:**
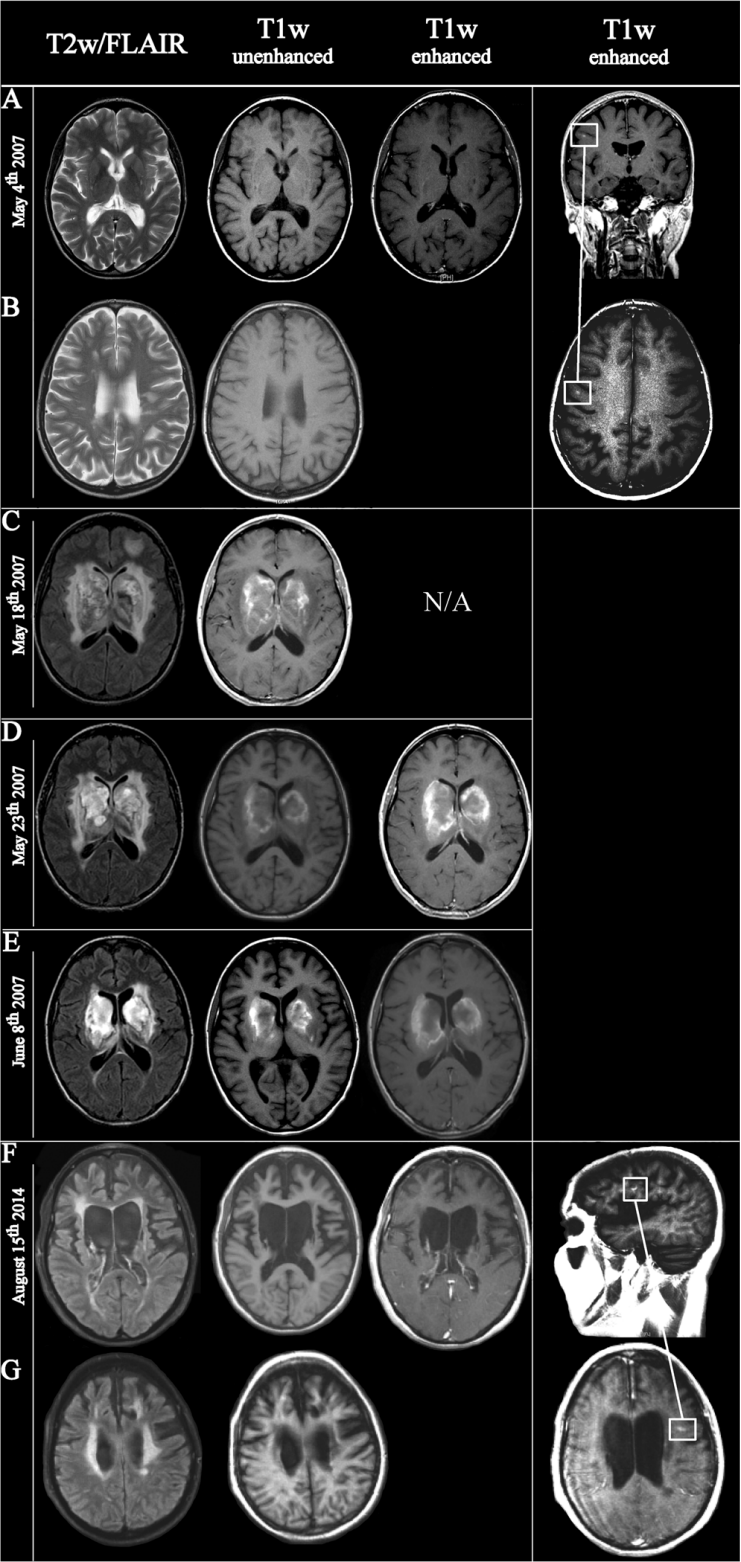
Chronological order of MRI scans from May 4th 2007 until August 15th 2014. **a** Images show normal deep nuclei and some unspecific small T2-weighted (T2w) hyperintensities. **b** The cranial T2w sequence depicts the high lesion load accompanied by several black holes in T1-weighted (T1w) sequences. Left-outermost column **a**–**b**. The *boxes* indicate a small nodular enhancement in the right precentral gyrus underlining disease progression. **c** The 2nd exam on May 18th, 2007 reveals the onset of space occupying haemorrhagic changes in the deep nuclei as well as hemorrhagic subcortical foci; hemorrhages are indicated by unenhanced T1w-hyperintensities. **d**–**e** The examinations from May 23rd und June 8th show a slow regression of the oedema and the size of the hemorrhagic areas. At both time points and in addition to the T1w signal hyperintensities caused by hemorrhagic residua, contrast enhancements can be found at the outer rim of the lesions. **f** By August 15th, 2014, only parenchymal defects are remaining together with distinctive brain volume loss leading to enlargement of ventricles. **g** Axial fluid attenuated inversion recovery imaging (FLAIR) shows an increase in lesions load. Both in the FLAIR and T1w image, confluent and mainly periventricular located lesions are found. Left-outermost column **f**–**g**. Boxes indicate a small enhancing focus in the left frontal hemisphere evidencing disease activity in multiple sclerosis. Images from May 5th, 2007 are with courtesy of Röntgenpraxis Georgstrasse, Hannover; images from August 15th, 2014 are with courtesy of Röntgenpraxis Marstall, Hannover. *N/A* not available

A then 21-year-old woman was diagnosed with relapsing-remitting MS in December 1993 according to the MS criteria of Poser et al. [5]. In 1996, she was enrolled in a clinical trial with glatiramer acetate and continued injecting this substance after the trial. After suffering relapses in 1997, 2001, 2004, and 2007, treatment was changed to interferon β-1a (Rebif® 44 μg three times a week) in April 2007. One month later, she developed numbness on the left side of her face. Magnetic resonance imaging (MRI) revealed a small contrast enhancing lesion in the right paracentral lobule (left-outermost column in Fig. 1a, b). Overall, the MRI depicted 23 supratentorial and one infratentorial lesion on T2-weighted scans underlining high lesion burden (Fig. 1b). A relapse was supposed and treatment with prednisolone 100 mg daily was initiated. High dose i.v. steroid treatment was not desired by the patient due to travel abroad. In preparation for her travel (Palma de Mallorca, Spain), she has not undergone any vaccination. After 4 days of treatment, the patient was admitted to the hospital while traveling because of progressive disturbance of consciousness.

Following intubation, the patient was transferred to the intensive care unit. The computed tomography (CT) showed hypodensities in the basal ganglia, frontal lobe, and mesencephalon. Internal venous thrombosis was ruled out by CT venous angiography; MRI revealed symmetrical hyperintense lesions in the basal ganglia on T1-weighted and punctate hemorrhages on T2-weighted images (Fig. 1c). Cerebrospinal fluid (CSF) analysis showed a normal cell count with 1 cell/μL and lactate levels within the normal range. There was an elevated protein indicating a severe dysfunction of the blood-CSF barrier (total protein 1.2 g/L). Initially, ceftriaxone, metronidazole, and cotrimoxazole were given i.v. on suspicion of meningoencephalitis.

**Fig. 2:**
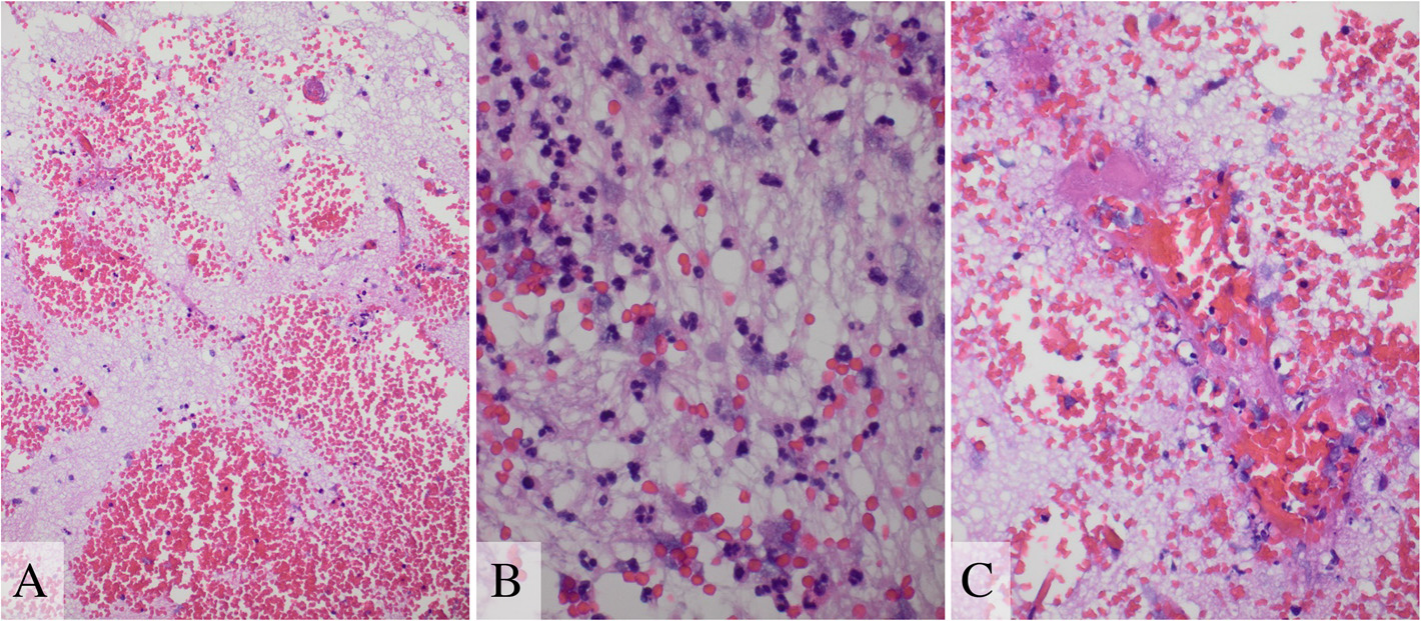
Hematoxylin and eosin staining. **a** Severe edema and various fresh bleedings predominately surround capillaries and small arterial vessels. **b** Neutrophilic infiltrates were found in some areas. The CNS tissue itself appeared necrotic. **c** Some smaller vessels exhibited a partial thrombosis

The patient developed seizures although MRI re-examination revealed a slight regression of the lesions in the basal ganglia (Fig. 1d). EEG showed generalized delta activity that was consistent with diffuse, severe encephalopathy. Chest X-ray and CT scans of the thorax and abdomen were normal. Serum and hematological laboratory examinations as well as blood cultures yielded unremarkable findings. Immunological parameters (ANA titer, rheumatoid factor, cyroglobulins) and toxicological screening of the blood and urine led to inconspicuous findings. Additional CSF analyses did not reveal evidence for infection with viruses (HSV, VZV, EBV, and CMV), *Borrelia burgdorferi*, syphilis, tuberculosis, *Cryptococcus*, and toxoplasmosis. There was no infection with HIV or hepatitis B and C. Thus, MRI-guided diagnostic brain biopsy of the parenchyma was performed. The predominant findings in the biopsy specimen were perivascular hemorrhagic necrosis and severe edema of the CNS tissue with an inflammatory infiltrate principally composed of neutrophils. Some of the vessels were partially thrombosed (Fig. 2). However, we did not observe a lymphocytic infiltration, macrophages, or astrocytic gliosis. Immunohistochemistry was negative for toxoplasma, HSV-1, HSV-2, EBV (LMP), and CMV. Therapy with intravenous methylprednisolone (1 g daily over 4 days, and then tapering of the dose) followed by intravenous immunoglobulins (30 g daily over 5 days) was initiated but without any effect. After 1 month, MRI showed a decline of lesion size and edema (Fig. 1e).

Clinically, the subject exhibited a severe spastic tetraparesis and was not able to speak. Thus, communication was only possibly using eye blinks or movements. Moreover, she experienced aching and rigidity in the extremities precipitated by tactile stimulation suggesting an abnormality in the long-latency stretch reflexes. The patient remained in this clinical state and had a stable clinical course on this poor level without a MS-specific therapy the following 7 years. In August 2014, the patient experienced a relapse that led to internuclear ophthalmoplegia. The most recent MRI examination displayed a new lesion located paramedian in the medulla oblongata explaining the disorder of conjugate lateral gaze. Moreover, we detected in the left frontal hemisphere a juxtacortically located small lesion with contrast uptake as well as a further one in the area of the middle cerebellar pedicle affecting both sides but without contrast uptake (left-outermost column in Fig. 1f, g; images that display the cerebellar pedicles are not shown). Intravenous methylprednisolone therapy (1 g daily over 5 days) led to a considerable improvement of the lateral gaze already at day 4 of administration. Taking the high EDSS of 9.5 into consideration, disease-modifying therapy was not initiated.

## Discussion

The present case demonstrates an unusual presentation during a classical course of MS leading to the diagnosis of AHLE. Diagnosis was provided by MRI findings showing symmetrical lesions of the basal ganglia and thalamus, histopathological analyses of affected tissue, and the rapid clinical deterioration.

AHLE, also known as Weston-Hurst disease, is characterized by acute and rapidly progressive inflammatory hemorrhagic demyelination of the white matter. The etiology remains unknown, but cross-reactivity between human myelin antigens and viral or bacterial antigens is supposed to induce an excessive immunological response which causes demyelination [6]. Recently, Pirko et al. demonstrated the first murine model of AHLE by injecting the VP2_121–130_ viral capsid of the Theiler’s murine encephalomyelitis virus that induces a strong in vivo activation of CD8^+^ T cells in C57BL/6 mice leading to the development of hemorrhagic demyelination within 24 h [7].

Acute necrotizing encephalopathy (ANE) might be another differential diagnosis in this case. It is a new disease entity proposed by Mizuguchi et al. in 1997 and is characterized by symmetrical, multifocal lesions, usually associated with edematous necrosis and hemorrhage, without inflammatory cells [8]. ANE always affects the thalami and, thus, causes a rapid disturbance of consciousness [9]. The etiology remains unknown, but a triggering viral infection is supposed. Point mutations in the RAN binding protein 2 and ephrin typ-B receptor 2 as a novel autoantigen are reported to be involved in disease mechanism [9].

The clinical differentiation between AHLE and ANE is a challenge because both diseases present similar findings on MRI and CSF. In this regard, the diagnostic criteria of ANE, proposed by Neilson et al. in 2010, might help to discern the appropriate diagnosis [8]. The crucial difference between ANE and AHLE is the invasion of inflammatory cells. In ANE, the pathologic hallmark is the absence of inflammatory cells, while in AHLE, neutrophilic infiltrates are detected almost regularly [8].

A literature search for similar cases was performed. We found a case of a 28-year-old woman who participated in a placebo-controlled trial of oral fingolimod in relapsing MS (FREEDOMS trial). She developed hemorrhaging focal encephalitis in the left temporoparietal white matter 7 months after commencing fingolimod [9]. A markedly increased disease activity was noticed since this patient developed several new T2 hyperintense lesions in the short-term disease course, one of which emerged in the left pulvinar thalami. This patient was diagnosed with tumefactive MS, but a brain biopsy was not performed [9]. In another case, a 40-year-old woman sustained a hemorrhagic lesion in the left frontal lobe during natalizumab treatment. The authors considered this as a secondary hemorrhage in a tumefactive lesion due to an increased vascular fragility. A biopsy of the lesion was not performed [10].

The term tumefactive MS refers to demyelinating lesions that are larger than those seen in “typical” MS, have an accompanying edema, and have mass effect resulting in symptoms and signs that are atypical for MS. Involvement of basal ganglia have been reported [11, 12]. In a biopsy series of 168 cases, Lucchinetti et al. observed hypercellular lesions with confluent demyelination, abundant foamy macrophages containing myelin debris, reactive astrogliosis, “relative” axonal preservation, and variable perivascular and parenchymal lymphocytic inflammation [11]. Moreover, astrocytic pleomorphism, astrocytes with fragmented nuclear inclusions (Creutzfelt–Peters cells), variable nuclear atypia, a rare mitotic figure, and occasional necrosis or cystic changes may histologically mimic a tumor [11]. However, Lucchinetti et al. did not report about hemorrhage in those lesions. According to the hypothesis forwarded by Kepes, such tumefactive lesions occupy an intermediate position between MS and ADEM, suggesting that the occurrence of AHLE in the present case would support this concept [13]. However, it is important to mention that before and after the AHLE episode, the subject had/has a rather “ordinary” MS course without any tumefactive or hemorrhagic lesions. We believe that the occurrence of AHLE was a separate event unrelated to MS.

AHLE and ADEM share many features since both are commonly preceded by upper respiratory infections or by HSV, EBV, rubella, measles, mumps, or influenza virus infection or vaccination even though overt symptoms of a preceding infection could not be found in the present case [6]. It remains elusive whether ADEM and AHLE may be part of a spectrum of disease with the same fundamental process resulting from an autoimmune process triggered by the prodromal infection, rather than distinct entities. However, different type of CNS infiltrates, i.e., lymphocytes in ADEM and neutrophils in AHLE, do not really support this idea of spectrum diseases.

In conclusion, the present case illustrates the uncommon presentation of AHLE in a MS patient. The diagnostic work-up is challenging, and brain biopsy offers guidance in the diagnosis of AHLE. The present case raises the question whether AHLE belongs to the spectrum of ADEM or displays a severe and discrete form of an immune response in the CNS.

## Consent

Written informed consent was obtained from the legal guardian of the patient for publication of this case report and any accompanying images. A copy of the written consent is available for review by the editor in chief of this journal.
